# Supplementary figures and images for: Dietary Supplementation of a Commercial Prebiotic, Probiotic and Their Combination Affected Growth Performance and Transient Intestinal Microbiota of Red Drum (Sciaenops ocellatus L.)
Source: Animals (Basel). 2022 Sep 30;12(19):2629. doi: 10.3390/ani12192629 (PMC9559286; doi:10.3390/ani12192629)

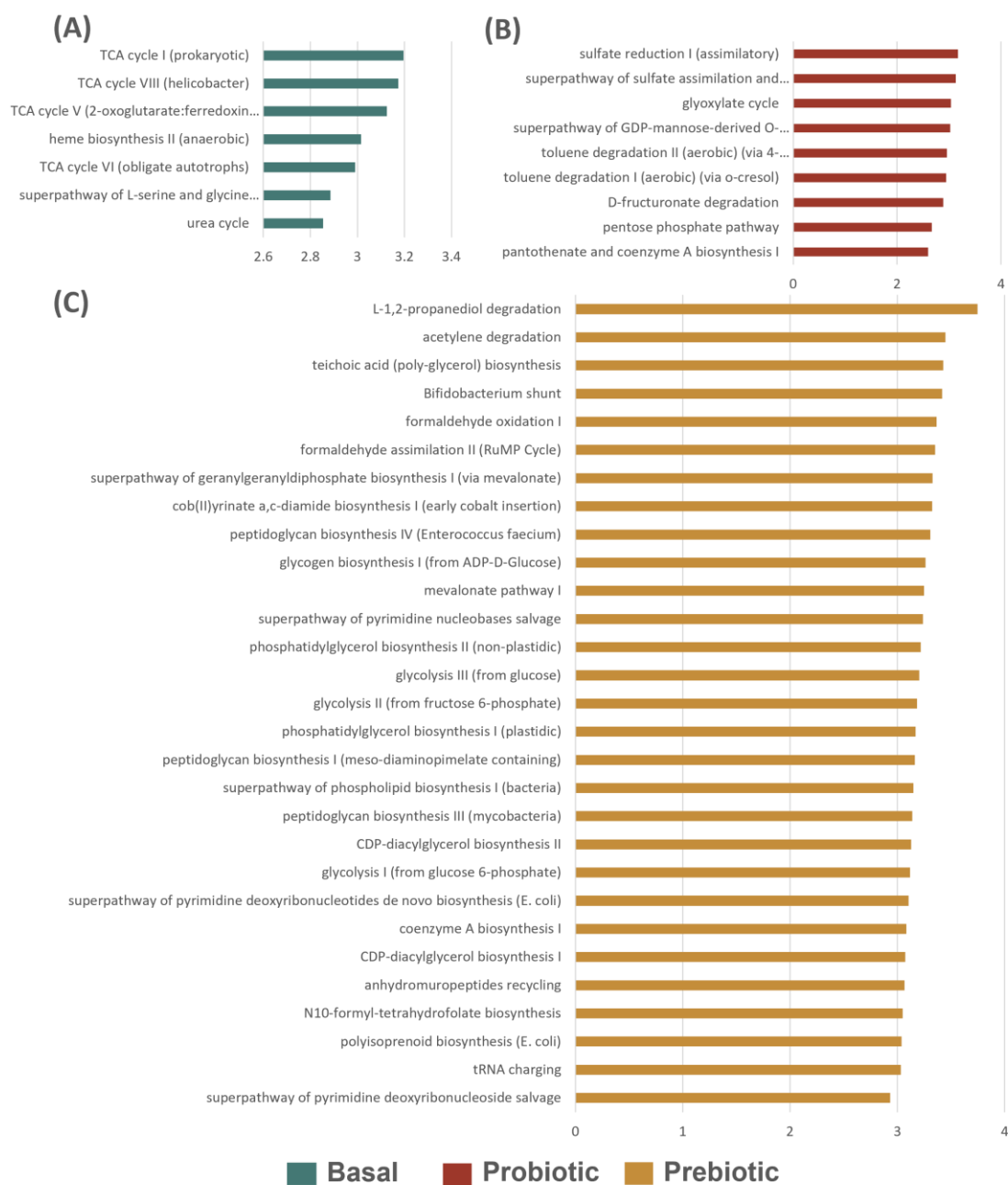

**Figure S1.** The remainder of the significant pathways. (a) Basal; (b) Probiotic; (c) Prebiotic.

Supplement: Supplementary file 1 [file animals-12-02629-s001.zip › animals-1850583-supplementary.pdf]
